# Preparation of Fluoroalkyl End-Capped Vinyltrimethoxysilane Oligomeric Silica Nanocomposites Containing Gluconamide Units Possessing Highly Oleophobic/Superhydrophobic, Highly Oleophobic/Superhydrophilic, and Superoleophilic/Superhydrophobic Characteristics on the Modified Surfaces

**DOI:** 10.3390/polym9070292

**Published:** 2017-07-20

**Authors:** Shinsuke Katayama, Shogo Fujii, Tomoya Saito, Shohei Yamazaki, Hideo Sawada

**Affiliations:** 1 Department of Frontier Materials Chemistry, Graduate School of Science and Technology, Hirosaki University, Hirosaki 036-8561, Japan; Shinsuke.Katayama@kantodenka.co.jp (S.K.); h15ms318@hirosaki-u.ac.jp (S.F.); hideosaw@cc.hirosaki-u.ac.jp (T.S.); shy@hirosaki-u.ac.jp (S.Y.); 2 Production Engineering Department, Kanto Denka Kogyo Co., Ltd., Kurashiki 712-8533, Japan

**Keywords:** fluoroalkyl end-capped oligomer, gluconamide unit, nanocomposite, gel, surface modification, highly oleophobic/superhydrophobic characteristic, highly oleophobic/superhydrophilic characteristic, superoleophilic/superhydrophobic characteristic

## Abstract

Fluoroalkyl end-capped vinyltrimethoxysilane oligomer [R_F_-(CH_2_-CHSi(OMe)_3_)*_n_*-R_F_ (R_F_-(VM)*_n_*-R_F_)] undergoes the sol-gel reaction in the presence of *N*-(3-triethoxysilylpropyl)gluconamide [Glu-Si(OEt)_3_] under alkaline conditions to afford the corresponding fluorinated oligomeric silica nanocomposites containing gluconamide units [R_F_-(VM-SiO_3/2_)*_n_*-R_F_/Glu-SiO_3/2_]. These obtained nanocomposites were applied to the surface modification of glass to provide the unique wettability characteristics such as highly oleophobic/superhydrophobic and highly oleophobic/superhydrophilic on the modified surfaces under a variety of conditions. Such a highly oleophobic/superhydrophobic characteristic was also observed on the modified PET (polyethylene terephthalate) fabric swatch, which was prepared under similar conditions, and this modified PET fabric swatch was applied to the separation membrane for the separation of the mixture of fluorocarbon oil and hydrocarbon oil. The R_F_-(VM-SiO_3/2_)*_n_*-R_F_/Glu-SiO_3/2_ nanocomposites, which were prepared under lower feed amounts of basic catalyst (ammonia), were found to cause gelation in water. Interestingly, it was demonstrated that these gelling nanocomposites are also applied to the surface modification of the PET fabric swatch to give a highly oleophobic/superhydrophobic characteristic on the surface. On the other hand, the modified glass surfaces treated with the corresponding nanocomposite possessing no gelling ability were found to supply the usual hydrophobic characteristic with a highly oleophobic property. More interestingly, the wettability change on the modified PET fabric swatch from highly oleophobic to superoleophilic was observed, and remained superhydrophobic after immersing the modified PET fabric swatch into water.

## 1. Introduction

It is well known that fluoroalkanoyl peroxide [R_F_-C(=O)OO(O=)C-R_F_: R_F_ = fluoroalkyl group] is a useful tool for the synthesis of two-fluoroalkyl end-capped oligomers [R_F_-(M)*_n_*-R_F_: M = radical polymerizable monomers] [1,2,3]. These fluoroalkyl end-capped oligomers are attractive polymeric materials because they can exhibit a variety of unique properties such as high solubility, surface active properties, and nanometer size-controlled self-assembled molecular aggregates through the aggregation of end-capped fluoroalkyl segments in oilgomers, which cannot be achieved by the corresponding non-fluorinated and randomly fluoroalkylated polymers [4,5]. In these fluoroalkyl end-capped oligomers, especially, fluoroalkyl end-capped oligomers containing various hydroxyl segments such as monool, triol, and tetraol can cause gelation in water and polar organic media such as methanol and ethanol, whose behavior is governed by the synergistic interactions of the aggregation of fluoroalkyl segments within oligomers and the intermolecular hydrogen bonding related to the hydroxyl segments [6,7,8,9]. In this way, the exploration of novel fluoroalkyl end-capped oligomers containing numerous hydroxyl segments is of particular interest, from the developmental viewpoints of new fluorinated functional materials. In a variety of hydroxylated polymers, much attention has been focused on the synthetic sugar-containing polymers (glycopolymers), which possess the pendent gluconic residues (pentaol units), owing to their role as biomimetic analogues and their applications in biomedical and technological fields such as paints and cosmetics [10,11,12,13,14,15,16,17,18,19]. In fact, poly(allylamine) can react with gluconolactone in water to provide the gluconamide-substituted polymers, successively affording the hydrogel through the interaction of the corresponding polymers with borax [20]. Similarly, the d-gluconamide unit-containing methacrylate monomer can be copolymerized with AIBN (2,2′-azobisisobutyronitrile) in the presence of methyl acrylate as comonomer to afford the corresponding glycopolymers [21]. These glycopolymers also have high potential use as polymeric surfactants [22]. Therefore, the studies on the preparation of novel gluconamide unit-containing polymers bearing longer fluoroalkyl groups are of particular interest, because these polymers would exhibit not only the unique characteristics related to the gluconamide units but also the surface active characteristics imparted by longer fluoroalky groups. However, such studies have been very limited so far. In our fluoroalkyl end-capped oligomers, especially, two fluoroalkyl end-capped vinyltrimethoxysilane oligomer [R_F_-(CH_2_-CHSi(OMe)_3_)*_n_*-R_F_] can undergo the sol-gel reaction under alkaline conditions to afford the corresponding fluorinated oligomeric silica nanoparticles [R_F_-(CH_2_-CHSiO_3/2_)*_n_*-R_F_] [22]. These fluorinated oligomeric silica nanoparticles have been also applied to the surface modification of glass to supply the superhydrophobic characteristic (water contact angle value: 180 degrees) with the oleophobic property [22]. From these findings, it is highly suggested that the composite reactions of our present fluorinated vinyltrimethoxysilane oligomer with the gluconamide derivatives should lead the obtained composites to the creation of the unique surface characteristics imparted by not only longer fluoroalkyl groups, but also the gluconamide units. Here we report that two fluoroalkyl end-capped vinyltrimethoxysilane oligomers [R_F_-(CH_2_–CHSi(OMe)_3_)*_n_*-R_F_] can undergo the sol-gel reaction in the presence of *N*-(3-triethoxysilylpropyl)gluconamide [Glu-Si(OEt)_3_] under alkaline conditions to afford the corresponding fluorinated oligomeric silica nanocomposites containing gluconamide units [R_F_-(VM-SiO_3/2_)*_n_*-R_F_/Glu-SiO_3/2_]. The modified surfaces treated with these obtained nanocomposites can provide the unique wettability states such as the highly oleophobic/superhydrophobic, highly oleophobic/superhydrophilic, and superoleophilic/superhydrophobic characteristics. These results will be described in this article.

## 2. Experimental

### 2.1. Materials

1,4-Bis[4-methylphenyl]amino]-9,10-anthracenedione (Quinizarin Green SS) and PET (polyethylene terephthalate) fabric (Polyester Tropical 13001) were received from Chugaikasei Co., Ltd. (Fukushima, Japan) and Unitika Ltd. (Osaka, Japan), respectively. *N*-(3-triethoxysilylpropyl)gluconamide [Glu-Si(OEt)_3_] was purchased from AZmax. Co. (Chiba, Tokyo). Dodecane and 1*H*-tridecafluorohexane were received from Tokyo Chemical Industrial Co., Ltd. (Tokyo, Japan). 25 wt % ammonia was provided by Wako Pure Chemical Industries (Osaka, Japan). Fluoroalkyl end-capped vinyltrimethoxysilane oligomer was prepared according to our previously reported method [23]. Glass plate (borosilicate glass) [micro cover glass: 18 mm × 18 mm] was purchased from Matunami glass Ind. Ltd. (Osaka, Japan) and was used after washing well with dichloromethane.

### 2.2. Measurements

Dynamic light scattering (DLS) measurements were measured by using Otsuka Electronics DLS-7000 HL (Tokyo, Japan). Contact angles were recorded using a Kyowa Interface Science Drop Master 300 (Saitama, Japan). Field emission scanning electron micrographs (FE-SEM) and energy dispersive X-ray (EDX) spectra were obtained by using JEOL JSM-7000F (Tokyo, Japan). Dynamic force microscope (DFM) was recorded by using SII Nano Technology Inc. E-sweep (Chiba, Japan).

### 2.3. Preparation of Fluoroalkyl End-Capped Vinyltrimethoxysilane Oligomeric Silica Nanocomposites Containing Gluconamide Units [R_F_-(VM-SiO_3/2_)_n_-R_F_/Glu-SiO_3/2_]

**Scheme 1:**
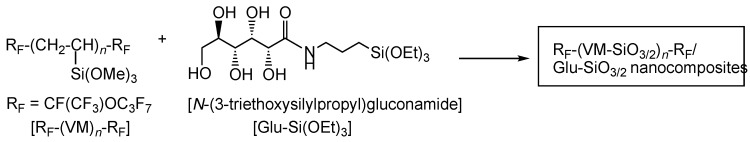
Preparation of R_F_-(VM-SiO_3/2_)*_n_*-R_F_/Glu-SiO_3/2_ nanocomposites.

A typical procedure for the preparation of nanocomposites is as follows: To methanol solution (5.0 mL) containing fluoroalkylated vinyltrimethoxysilane oligomer [200 mg; R_F_-(CH_2_CHSi(OMe)_3_)*_n_*-R_F_; R_F_ = CF(CF_3_)OC_3_F_7_; M_n_ = 730 (R_F_-(VM)*_n_*-R_F_)] was added 50 wt % Glu-Si(OEt)_3_ ethanol solution (1400 mg). The mixture was stirred with a magnetic stirring bar at room temperature for 30 min. 25% aqueous ammonia solution (1.0 mL) was added to this mixture, and then stirred for 5 h at room temperature. Methanol was added to the obtained crude products after the solvent was evaporated off. The methanol suspension was stirred with magnetic stirring bar at room temperature for 1 day. The fluorinated oligomeric silica/Glu-SiO_2_ nanocomposites were isolated after centrifugal separation for 30 min. The nanocomposite product was washed well with methanol several times, and then was dried under vacuum at 50 °C for 1 day to afford the expected nanocomposites as white powders (774 mg) (see Scheme 1 and Table 1).

### 2.4. Preparation of the Modified Glass Treated with the R_F_-(VM-SiO_3/2_)_n_-R_F_/Glu-SiO_3/2_ Nanocomposites by Dipping Method

To methanol solution (5.0 mL) containing R_F_-(VM)*_n_*-R_F_ oligomer (200 mg) was added 50 wt % Glu-Si(OEt)_3_ ethanol solution (700 mg) and 25% aqueous ammonia solution (1.0 mL). The mixture was stirred with a magnetic stirring bar at room temperature for 5 h. The glass plates (18 × 18 mm^2^ pieces) were dipped into this methanol solution at room temperature and left for 1 min. The glass plate was lifted from the solutions at a constant rate of 0.5 mm/min and were left to dry at room temperature for 1 day; finally, these were dried under vacuum for 1 day at room temperature to afford the modified glass. The modified PET fabric swatch (25 × 25 mm^2^ pieces) was prepared under similar conditions. The contact angle values for dodecane and water were measured by depositing a drop of dodecane (2 µL) or water (2 µL) on the modified plate surfaces.

## 3. Results and Discussion

Sol-gel reaction of fluoroalkyl end-capped vinyltrimethoxysilane oligomer [R_F_-(CH_2_CHSi(OMe)_3_)*_n_*-R_F_; R_F_ = CF(CF_3_)OC_3_F_7_ (R_F_-(VM)*_n_*-R_F_)] proceeded smoothly in the presence of *N*-(3-triethoxysilylpropyl)gluconamide [Glu-Si(OEt)_3_] under alkaline conditions to afford the corresponding fluorinated oligomeric silica/Glu-SiO_3/2_ nanocomposites [R_F_-(VM-SiO_3/2_)*_n_*-R_F_/Glu-SiO_3/2_]. These results are shown in Scheme 1 and Table 1.

The expected fluorinated composites were obtained in 31–86% isolated yields, and the obtained composites revealed a good dispersibility and stability in traditional organic media such as methanol, tetrahydrofuran, and 1,2-dichloroethane. Especially the fluorinated composites (Runs: 1–5), which were prepared under the feed amounts of Glu-Si(OEt)_3_ from 10 to 170 mg, afforded no dispersibility toward water; however, the fluorinated composites (Runs: 6–8), which were prepared under a higher feed amount of Glu-Si(OEt)_3_ greater than 170 mg, were found to give a good dispersibility and stability toward water.

The size of the fluorinated composites in Table 1 was measured in methanol by dynamic light-scattering (DLS) measurements at 25 °C, because these composites can reveal the good dispersibility in methanol. Table 1 also shows that each size of the composites is nanometer size-controlled fine particles from 33 to 72 nm (number-average diameter).

**Figure 1:**
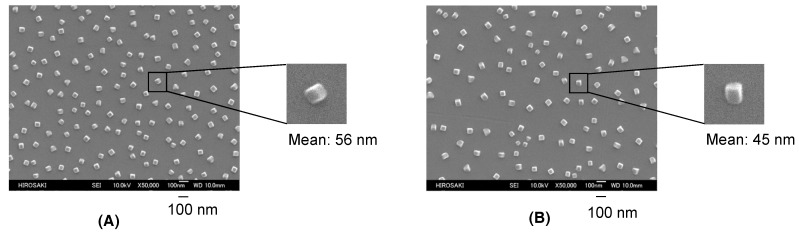
FE-SEM (Field Emission Scanning Electron Microscopy) images of well-dispersed methanol solutions of the R_F_-(VM-SiO_3/2_)*_n_*-R_F_/Glu-SiO_3/2_ nanocomposite particles: (**A**): Run 4; (**B**): Run 7 in Table 1.

FE-SEM photograph of the R_F_-(VM-SiO_3/2_)*_n_*-R_F_/Glu-SiO_3/2_ nanocomposites (Runs 4 and 7 in Table 1) methanol solutions was recorded, and the results are shown in Figure 1.

Electron micrograph also shows the formation of fluorinated nanocomposite fine cubic-type particles with a mean diameter of 56 and 45 nm, respectively, and the similar size values to those (37.4 ± 9.0 and 51.1 ± 6.5 nm) of DLS measurements were observed in FE-SEM measurements (see Runs 4 and 7 in Table 1).

To verify the surface active characteristics of the nanocomposites in Table 1, these fluorinated nanocomposites have been applied to the surface modification of glass, and we have measured the dodecane and water contact angle values on these modified surfaces. The results are shown in Table 2.

As shown in Table 2, water contact values are, in general, dependent upon the feed ratios of the R_F_-(VM)*_n_*-R_F_ oligomer and the Glu-Si(OEt)_3_ employed, increasing with lower feeds amounts of Glu-Si(OEt)_3_ in R_F_-(VM)*_n_*-R_F_/Glu-(Si(OEt)_3_ from 108 to 180 degrees (superhydrophobic surface). A similar result was observed in the dodecane contact angle values, increasing from 56 to 112 degrees except for Run 1, although the original fluoroalkyl end-capped vinyltrimethoxysilane oligomeric silica nanoparticles [R_F_-(VM-SiO_3/2_)*_n_*-R_F_] can give a usual oleophobic (dodecane contact angle value: 46 degrees) with a superhydrophobic property (water contact angle value: 180 degrees).

The water contact angle values on the modified glass surfaces treated with the fluorinated nanocomposites, which were prepared under the greater feed amounts of Glu-Si(OEt)_3_ from 0.44 to 1.75 mmol, were found to decrease from 133–137 to 0 degrees (superhydrophilic surface) over 15 or 25 min except for Run 8, indicating that the hydrophobic fluoroalkyl segments are replaced by the hydrophilic gluconamide segments in the nanocomposites when the environment is changed from air to water. The relatively higher feed amounts (0.44–0.88 mmol) of Glu-Si(OEt)_3_ in the nanocomposite preparation illustrated in Scheme 1 would enable the smooth surface arrangement of the hydrophilic gluconamide segments to provide the completely superhydrophilic surface through the flip-flop motion between the fluoroalkyl groups and gluconamide segments in the nanocomposites. A similar flip-flop motion between longer fluoroalkyl groups and hydrophilic segments has been already reported to exhibit the hydrophilic characteristic with an oleophobic property on the modified surfaces [24,25,26,27,28]. Therefore, the nanocomposites which were prepared under relatively lower feed amounts (0.03 mmol) of Glu-Si(OEt)_3_, can supply a highly oleophobic/superhydrophobic characteristic (dodecane and water contact angle values: 112 and 180 degrees) on the modified surface, due to the lower content of hydrophilic gluconamide segments in the nanocomposites.

**Figure 2:**
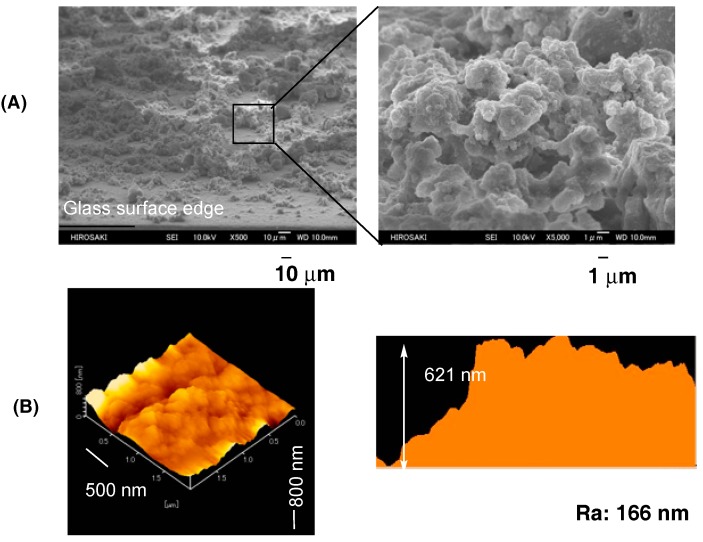
FE-SEM (Field Emission Scanning Electron Microscopy) images (**A**) and DFM (Dynamic Force Microscopy) topography (**B**) of the modified glass surface treated with the R_F_-(VM-SiO_3/2_)*_n_*-R_F_/Glu-SiO_3/2_ nanocomposites (Run 2 in Table 1).

**Figure 3:**
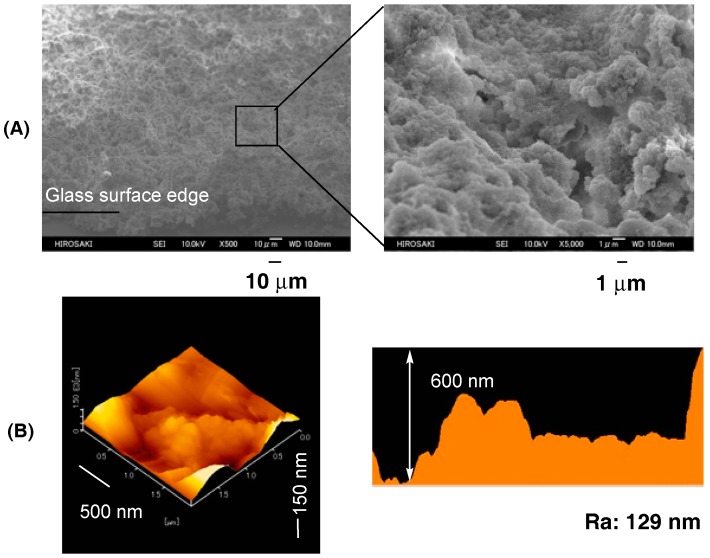
FE-SEM (Field Emission Scanning Electron Microscopy) images (**A**) and DFM (Dynamic Force Microscopy) topography (**B**) of the modified glass surface treated with the R_F_-(VM-SiO_3/2_)*_n_*-R_F_/Glu-SiO_3/2_ nanocomposites (Run 1 in Table 1).

**Figure 4:**
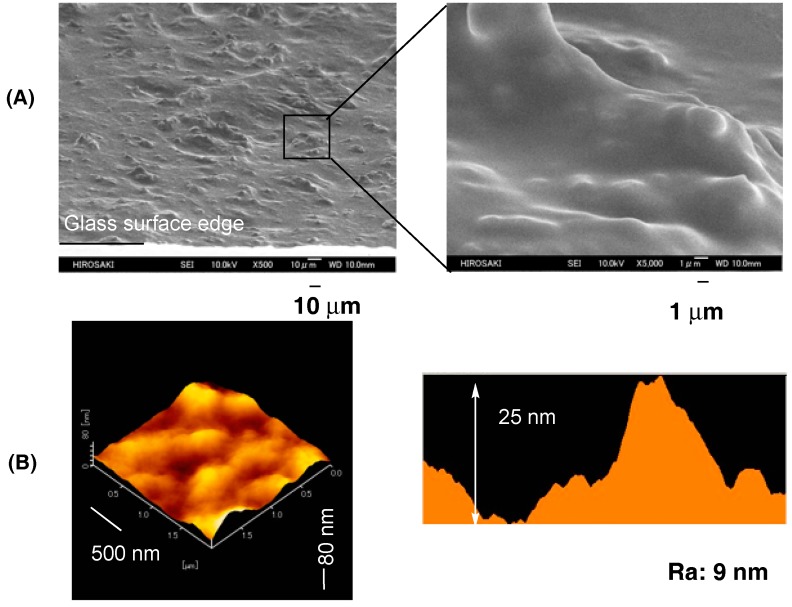
FE-SEM (Field Emission Scanning Electron Microscopy) images (**A**) and DFM (Dynamic Force Microscopy) topography (**B**) of the modified glass surface treated with the R_F_-(VM-SiO_3/2_)*_n_*-R_F_/Glu-SiO_3/2_ nanocomposites (Run 8 in Table 1).

**Figure 5:**
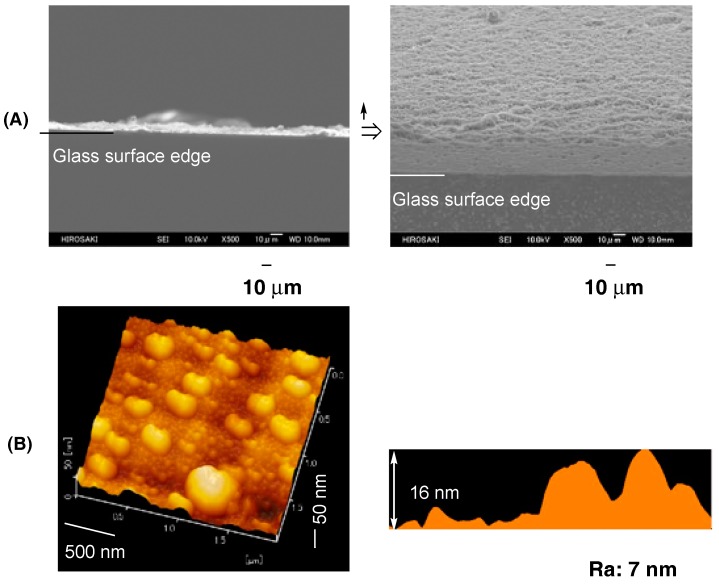
FE-SEM (Field Emission Scanning Electron Microscopy) images (**A**) and DFM (Dynamic Force Microscopy) topography (**B**) of the modified glass surface treated with the pristine R_F_-(VM-SiO_3/2_)*_n_*-R_F_/Glu-SiO_3/2_ nanocomposites (see Table 1).

The fabrication of a superoleophobic surface is, in general, difficult due to the lower surface tension of oils than that of water. Thus, a highly oleophobic (superoleophobic) surface can be realized by lowering the surface energy and enhancing the surface roughness [29,30,31,32,33]. Similarly, it is well known that a superhydrophobic surface can be created by the architecture of the roughness surface through the sol-gel reactions of the longer alkyl chain-containing silane coupling agents such as octadecyltrichlorosilane and hexadecyltriethoxysilane in the presence of silica nanoparticles and tetraethoxysilane under alkaline conditions [34,35]. Therefore, in order to clarify such unique surface wettability illustrated in Table 2, we have studied the surface roughness of the modified glasses treated with the R_F_-(VM-SiO_3/2_)*_n_*-R_F_/Glu-SiO_3/2_ nanocomposites possessing the highly oleophobic/superhydrophobic characteristic (Run 2 in Table 2: dodecane contact angle value: 112 degrees and water contact angle value: 180 degrees) by using FE-SEM (field emission scanning electron microscopy) measurements and DFM (dynamic force microscopy) measurements, respectively. We have also studied the surface roughness of the modified surfaces possessing a similar wettability (highly oleophobic/superhydrophobic characteristic) (Run 1 in Table 2) and usual oleophobic and hydrophobic characteristics (Run 8 in Table 2) including the original fluoroalkyl end-capped vinyltrimethoxysilane oligomeric silica nanoparticles [R_F_-(VM-SiO_3/2_)*_n_*-R_F_], for comparison. The results are shown in Figure 2, Figure 3, Figure 4 and Figure 5.

As shown in Figure 2A, the architecture of the effective roughness was observed on the modified surface treated with the R_F_-(VM-SiO_3/2_)*_n_*-R_F_/Glu-SiO_3/2_ nanocomposites possessing a highly oleophobic/superhydrophobic property. Such roughness of surface was also observed in the nanocomposites possessing a similar wettability depicted in Figure 3A. The topographical image of the modified glass surface treated with the nanocomposites possessing a highly oleophobic/superhydrophobic property (Figure 2B) can afford a higher roughness characteristic (the roughness average value: Ra value = 166 nm), compared with that (Ra value = 129 nm) of the modified surface possessing a lower dodecane contact angle value: 74 degrees (see Figure 3B). Such a higher Ra value should enable the modified surface to supply a highly oleophobic/superhydrophobic characteristic. Especially, the introduction of an air cushion into rough deposits of our present nanocomposite particles could create such a unique superoleophobic/superhydrophobic characteristic on the modified surface. In fact, it was reported that the introduction of a proper rough surface microstructure could make a flat hydrophobic surface more hydrophobic due to the introduction of an air cushion beneath the water droplet [36]. On the other hand, the FE-SEM and DFM measurements (see Figure 4 and Figure 5) show that the modified surfaces treated with the R_F_-(VM-SiO_3/2_)*_n_*-R_F_/Glu-SiO_3/2_ nanocomposites possessing a usual oleophobic/hydrophobic property and the pristine R_F_-(VM-SiO_3/2_)*_n_*-R_F_ oligomeric nanoparticles can provide relatively smooth roughness, because these Ra values are 9 and 7 nm, respectively, indicating that such relatively smooth surfaces should supply the usual oleophobic/hydrophobic or oleophobic/superhydrophobic characteristic to the modified surfaces.

**Figure 6:**
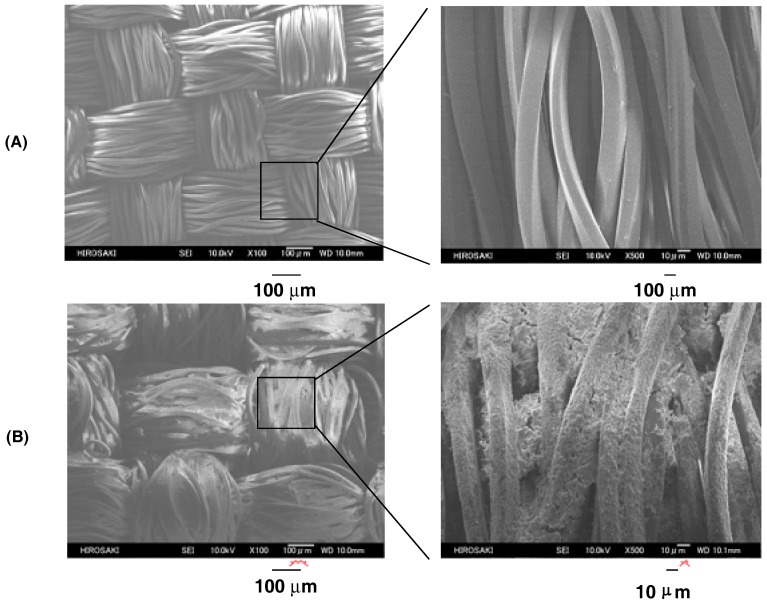
FE-SEM (Field Emission Scanning Electron Microscopy) images of the pristine PET-fabric swatch (**A**) and the modified PET fabric swatch (**B**) treated with the R_F_-(VM-SiO_3/2_)*_n_*-R_F_/Glu-SiO_3/2_ nanocomposites (Run 2 in Table 1).

**Figure 7:**
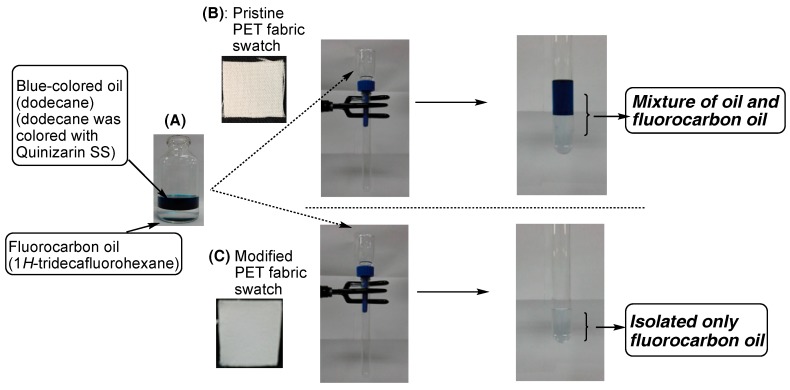
Separation of the mixture (**A**) of the blue-colored hydrocarbon oil (dodecane) and fluorocarbon oil (1*H*-tridecafluorohexane) by using the pristine PET fabric swatch (**B**) and the modified PET fabric swatch treated with the R_F_-(VM-SiO_3/2_)*_n_*-R_F_/Glu-SiO_3/2_ nanocomposites (Run 2 in Table 1) (**C**) as the separation membrane, respectively.

We tried to achieve the surface modification of not only the glass but also the polyethylene terephthalate (PET) fabric swatch by using the R_F_-(VM-SiO_3/2_)*_n_*-R_F_/Glu-SiO_3/2_ nanocomposites (Run 2 in Table 1) under similar conditions, and the dodecane and water contact angle values on the modified PET fabric swatch were measured. The modified PET fabric swatch can exhibit the similar dodecane and water contact angle values: 111 and 180 degrees to those of the modified glass surfaces depicted for Run 2 in Table 2. Especially, FE-SEM pictures of the modified PET fabric swatch show that the fluorinated nanocomposite particles are not only filled up between the PET fibers, but are also uniformly coated on the PET fibers (Figure 6B), compared with that of the pristine PET fabric (Figure 6A). Here, we tried to apply this modified PET fabric swatch to the separation membrane for the separation of the mixture of the fluorocarbon oil (1*H*-tridecafluorohexane) and the hydrocarbon oil (dodecane: blue-colored with Quinizarin Green SS), because the modified PET fabric swatch can provide a highly oleophobic/superhydrophobic characteristic on the modified surface, and the results are illustrated in Figure 7.

As shown in Figure 7C, the surface appearance of the modified PET fabric swatch was quite similar to that of the pristine PET fabric swatch Figure 7B. In addition, it was demonstrated that the modified PET fabric swatch is effective for the separation of blue-colored hydrocarbon oil and fluorocarbon oil to isolate the transparent colorless fluorocarbon oil as shown in Figure 7C; although the pristine PET fabric swatch was not able to separate the mixture under similar conditions (see Figure 7B). This finding is due to the highly oleophobic/superhydrophobic characteristic of the modified PET fabric swatch.

As illustrated in Scheme 1 and Table 1, the expected fluorinated oligomeric silica nanocomposites containing gluconamide units are prepared through the sol-gel reactions under alkaline conditions. Especially, as mentioned before, the higher feed amounts of Glu-Si(OEt)_3_ greater than 0.21 mmol can lead the obtained nanocomposites to a good dispersibility toward water. This finding suggests that the fluorinated nanocomposites can cause gelation toward water through the synergistic interaction between the aggregation of the end-capped fluoroalkyl groups and the intermolecular hydrogen bonding related to the hydroxyl segments in the gluconamide units in the nanocomposites by controlling the sol-gel conditions (for example; by changing the alkaline concentrations). In fact, we have succeeded in preparing the R_F_-(VM-SiO_3/2_)*_n_*-R_F_/Glu-SiO_3/2_ nanocomposites, which can cause gelation toward water, by changing the feed amounts of both 25 % aqueous ammonia and methanol from 1.0 to 0.6 and 5.0 to 15 mL, respectively (see Table 3).

**Figure 8:**
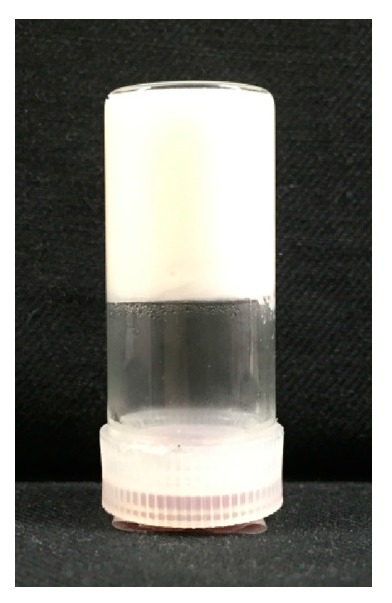
Gelation of water by using the R_F_-(VM-SiO_3/2_)*_n_*-R_F_/Glu-SiO_3/2_ nanocomposites (Run 9 in Table 3).

The gelling fluorinated nanocomposites (see Figure 8) were applied to the surface modification of the PET fabric swatch under similar conditions to those of Table 2, and we have measured the dodecane and water contact angle values on the modified PET fabric swatch. The results are shown in Table 4.

**Figure 9:**
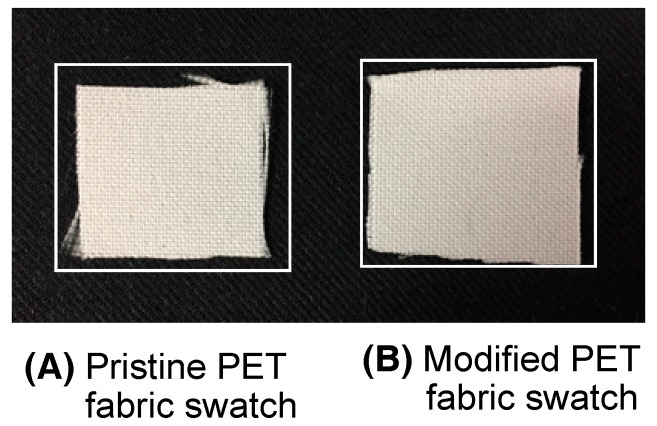
Photograph of the pristine PET fabric swatch (**A**) and the modified PET fabric swatch (**B**) treated with the R_F_-(VM-SiO_3/2_)*_n_*-R_F_/Glu-SiO_3/2_ nanocomposites gels (Run 9 in Table 3).

**Figure 10:**
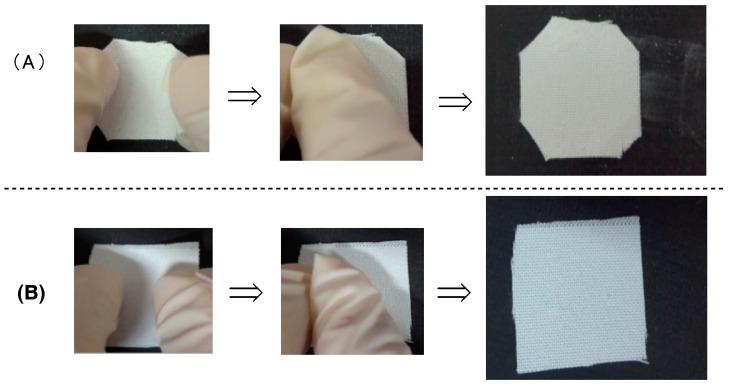
Photograph of the adhesion trial for the modified PET fabric swatch treated with the pristine R_F_-(VM-SiO_3/2_)*_n_*-R_F_ oligomeric nanoparticle (**A**) and the modified PET fabric swatch treated with the R_F_-(VM-SiO_3/2_)*_n_*-R_F_/Glu-SiO_3/2_ nanocomposite gels (Run 9 in Table 3) (**B**).

**Figure 11:**
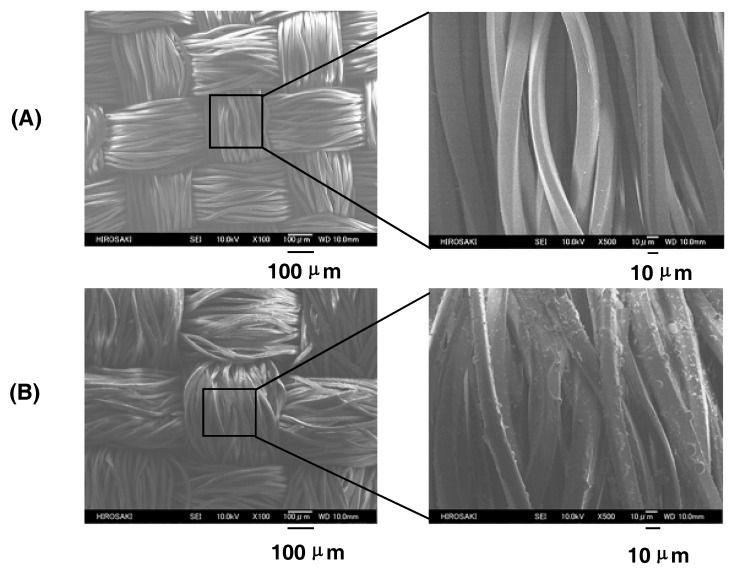
FE-SEM (Field Emission Scanning Electron Microscopy) images of the pristine PET fabric swatch (**A**) and the modified PET fabric swatch treated with the R_F_-(VM-SiO_3/2_)*_n_*-R_F_/Glu-SiO_3/2_ nanocomposite gels (Run 9 in Table 3) (**B**).

As shown in Table 4, the modified PET fabric swatches were found to give a highly oleophobic/superhydrophobic characteristic, because the dodecane and water contact angle values are 89–102 and 180 degrees, respectively, quite different from the corresponding modified glass surfaces possessing a highly oleophobic/hydrophobic characteristic (see Runs 4 and 5 in Table 2). The surface appearance of the modified PET fabric swatch is almost the same as that of the pristine PET fabric swatch (see Figure 9), and the adhesion ability of the PET fabric swatch is strong enough that even after rubbing the modified surface with a finger, we cannot detect any released nanocomposites powder (see Figure 10B). In contrast, the adhesion ability of the modified PET fabric swatch treated with the pristine R_F_-(VM-SiO_3/2_)*_n_*-R_F_ oligomeric nanoparticles is not enough, and the corresponding fluorinated oligomeric nanoparticles were easily released from the modified surface after rubbing the surface with a finger (see Figure 10A). Such strong adhesion ability would be due to the uniform modification on the PET fiber surface by the R_F_-(VM-SiO_3/2_)*_n_*-R_F_/Glu-SiO_3/2_ nanocomposite gels. In fact, FE-SEM images of the modified PET fabric and the pristine PET fabric swatches show that the nanocomposite gels can be uniformly coated on the PET fibers (see Figure 11A,B), quite different from the FE-SEM picture of the modified PET fabric treated with the R_F_-(VM-SiO_3/2_)*_n_*-R_F_/Glu-SiO_3/2_ nanocomposites possessing no gelling ability as in Figure 6B.

Furthermore, we have measured the dodecane and water contact angle values on the modified PET fabric swatches treated with the nanocomposite gels (Runs 9 and 10 in Table 4) after immersing the corresponding swatches in water at room temperature for 1 day, and the results are also shown in Table 4.

**Figure 12:**
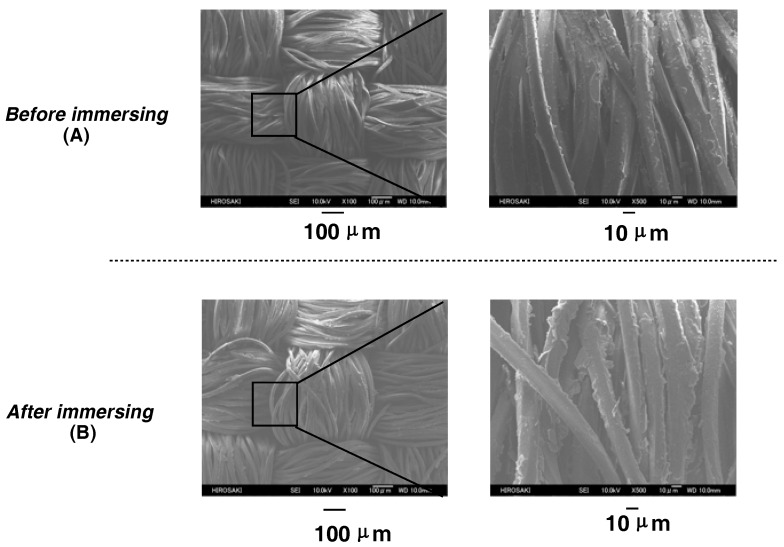
FE-SEM (Field Emission Scanning Electron Microscopy) images of the modified PET fabric swatch treated with the R_F_-(VM-SiO_3/2_)*_n_*-R_F_/Glu-SiO_3/2_ nanocomposites gels (Run 9 in Table 2) before (**A**) and after (**B**) immersing into water at room temperature for 1 day.

Quite interestingly, we can observe the wettability change from the highly oleophobic to the superoleophilic characteristic, while keeping the superhydrophobic characteristic on the modified fabric swatches only after immersing into water, because the dodecane contact angle values extremely decreased from 89–102 to 0 degrees after immersing into water. We have measured the FE-SEM images of the modified PET fabric swatch after immersing into water, and the results are illustrated in Figure 12B. The FE-SEM images show that the nanocomposite gels are uniformly coated on each PET fiber to enhance the roughness on the PET fibers, different from that before immersing into water (see Figure 12A). Such enhanced roughness would afford the different wettability through the immersing process into water.

**Figure 13:**
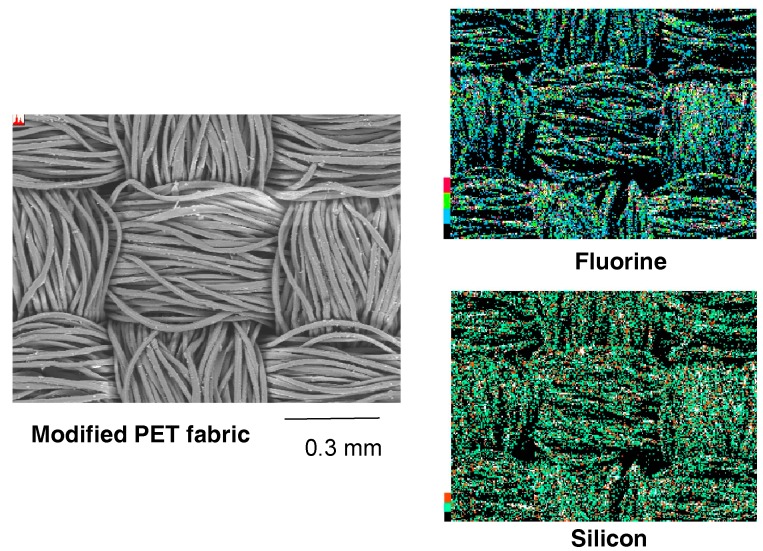
EDX (Energy Dispersive X-ray) mapping micrographs of the fluorine and silicon atoms on the modified PET fabric swatch surface treated with the R_F_-(VM-SiO_3/2_)*_n_*-R_F_/Glu-SiO_3/2_ nanocomposite gels (Run 9 in Table 3) before immersing into water.

**Figure 14:**
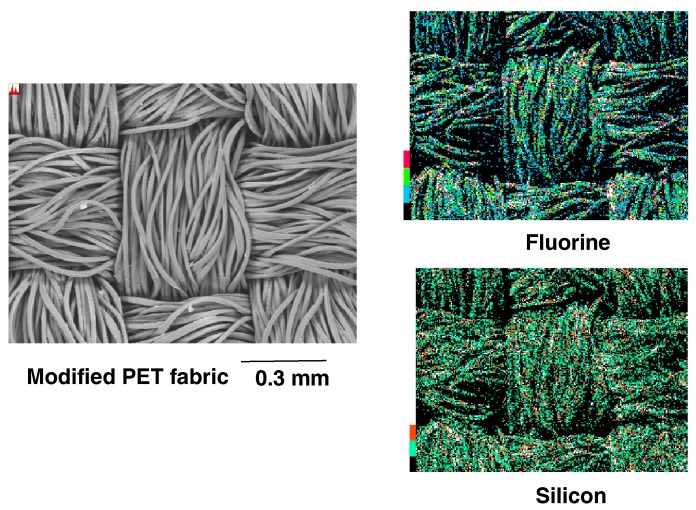
EDX (Energy Dispersive X-ray) mapping micrographs of the fluorine and silicon atoms on the modified PET fabric swatch surface treated with the R_F_-(VM-SiO_3/2_)*_n_*-R_F_/Glu-SiO_3/2_ nanocomposite gels (Run 9 in Table 3) after immersing into water at room temperature for 1 day.

In order to clarify the presence of fluorinated nanocomposites in the modified PET fabric swatch even after immersing into water, we have measured the EDX (Energy Dispersive X-ray) spectra of the modified PET fabric swatch treated with the R_F_-(VM-SiO_3/2_)*_n_*-R_F_/Glu-SiO_3/2_ nanocomposites (Runs 9 and 10 in Table 4) before and after immersing into water, the results are shown in Figure 13 and Figure 14.

EDX measurements show that the atomic contents of carbon, oxygen, nitrogen, fluorine, and silicon of the modified PET fabric swatch before and after immersing into water are as follows (see Table 5).

We observe similar atomic values even after immersing into water. In addition, it was verified that fluorine and silicon atoms related to the fluorinated nanocomposites are uniformly dispersed on the modified PET fabric swatch before and after immersing into water by EDX mapping images illustrated in Figure 13 and Figure 14.

In this way, it was demonstrated that our present R_F_-(VM-SiO_3/2_)*_n_*-R_F_/Glu-SiO_3/2_ nanocomposite gels can be strongly coated into the PET fabric fiber networks due to the gelling ability of the nanocomposites.

## 4. Conclusions

Fluoroalkyl end-capped viniyltrimethoxysilane oligomeric silica nanocomposites containing gluconamide units [R_F_-(VM-SiO_3/2_)*_n_*-R_F_/Glu-SiO_3/2_] were prepared by the sol-gel reactions of the corresponding oligomers in the presence of gluconamide unit-containing silane coupling agent [Glu-Si(OEt)_3_] under alkaline conditions. Wettability control between the highly oleophobic/superhydrophobic and highly oleophobic/superhydrophilic states was observed on the modified glass surfaces treated with the R_F_-(VM-SiO_3/2_)*_n_*-R_F_/Glu-SiO_3/2_ nanocomposites. Such wettability can be easily controlled by changing the feed amount ratios of Glu-Si(OEt)_3_ and R_F_-(VM)*_n_*-R_F_ oligomer for the preparation of the nanocomposites. Lower feed amounts of Glu-Si(OEt)_3_ can provide a highly oleophobic/superhydrophobic surface; in contrast, a higher feed amount of Glu-Si(OEt)_3_ enables the modified surface to reveal a highly oleophobic/superhydrophilic characteristic. Such a highly oleophobic/superhydrophobic characteristic was also observed on the modified PET fabric swatch, and this modified PET fabric was applied to the separation membrane to separate the mixture of fluorocarbon oil and hydrocarbon oil.

R_F_-(VM-SiO_3/2_)*_n_*-R_F_/Glu-SiO_3/2_ nanocomposites were also shown to cause gelation toward water. Especially, the gelling nanocomposites were applied to the surface modification of the PET fabric swatch to exhibit a highly oleophobic/superhydrophobic characteristic on the modified surface. However, interestingly, we can observe the wettability change on the modified PET fabric surface from a highly oleophobic state to a superoleophilic state, while keeping the superhydrophobic characteristic by immersing this modified fabric into water.

## Figures and Tables

**Table 1:** Preparation of the R_F_-(VM-SiO_3/2_)*_n_*-R_F_/Glu-SiO_3/2_ nanocomposites.

Run	R_F_-(VM)*_n_*-R_F_ (mg) (mmol)	Glu-Si(OEt)_3_ (mg) ^c^ (mmol)	MeOH (mL)	25 wt % aq. NH_3_ (mL)	Yield ^a^ (%)	Size of Composites ^b^ (nm ± STD)
1	200 (0.27)	10 (0.01)	5.0	1.0	54	32.7 ± 3.1
2	200 (0.27)	25 (0.03)	5.0	1.0	39	38.1 ± 7.1
3	200 (0.27)	50 (0.06)	5.0	1.0	43	53.8 ± 17.3
4	200 (0.27)	90 (0.11)	5.0	1.0	39	37.4 ± 9.0
5	200 (0.27)	170 (0.21)	5.0	1.0	31	71.8 ± 14.0
6	200 (0.27)	350 (0.44)	5.0	1.0	43	35.5 ± 2.3
7	200 (0.27)	700 (0.88)	5.0	1.0	69	51.1 ± 6.5
8	200 (0.27)	1400 (1.75)	5.0	1.0	86	42.8 ± 8.7

^a^ Yield was based on oligomer and Glu-Si(OEt)_3_; ^b^ Determind by dynamic light scattering (DLS) measurements in methanol; ^c^ 50% EtOH solution.

**Table 2:** Contact angles of dodecane and water on the modified glasses treated with the R_F_-(VM-SiO_3/2_)*_n_*-R_F_/Glu-SiO_3/2_ nanocomposites.

Run *	**Feed Ratio (mmol/mmol) (R_F_-(VM)-R_F_/Glu-Si(OEt)_3_)**	**Dodecane**	**Contact Angle (Degree)**						
			**Water**						
			**Time**						
			0 m	5 m	10 m	15 m	20 m	25 m	30 m
1	(0.27/0.01)	74	180	- **	-	-	-	-	-
2	(0.27/0.03)	112	180	- **	-	-	-	-	-
3	(0.27/0.06)	107	142	130	132	131	128	127	104
4	(0.27/0.11)	97	137	134	134	129	127	127	122
5	(0.27/0.21)	94	135	133	132	132	128	113	101
6	(0.27/0.44)	97	137	143	122	91	62	0	- **
7	(0.27/0.88)	58	133	132	104	0	- **	-	-
8	(0.27/1.75)	56	108	106	102	99	93	86	80
Parent R_F_-(VM-SIO_2_)*_n_*-R_F_		46	180	- **	-	-	-	-	-
Non-treated glass		0	50						

* Each Run No corresponds to that of Table 1; ** no change.

**Table 3:** The feed amounts of R_F_-(VM)*_n_*-R_F_ oligomer, Glu-Si(OEt)_3_, aqueous ammonia and methanol for the preparation of the gelling R_F_-(VM-SiO_3/2_)*_n_*-R_F_/Glu-SiO_3/2_ nanocomposites.

Run No.	Feed Amounts (mg/mg) of		
	R_F_-(VM)*_n_*-R_F_/Glu-Si(OEt)_3_	25% aq. Ammonia (mL)	Methanol (mL)
9	200/90 [0.27/0.11(mmol/mmol)]	0.6	15
10	200/170 [0.27/0.21 (mmol/mmol)]	0.6	15

**Table 4:** Contact angles of dodecane and water on the modified PET fabrics swatch treated with the R_F_-(VM-SiO_3/2_)*_n_*-R_F_/Glu-SiO_3/2_ nanocomposite gels before and after immersing into water at room temperature for 1 day.

**Run**	**Feed Ratio (mmol/mmol) (R_F_-(VM)-R_F_/Glu-Si(OEt)_3_)**	**Dodecane**	**Contact Angle (Degree)**						
			**Water**						
			**Time (min)**						
			0 m	5 m	10 m	15 m	20 m	25 m	30 m
Before immersing into water									
9	(0.27/0.11)	102	180	- ^a^	-	-	-	-	-
10	(0.27/0.21)	89	180	- ^a^	-	-	-	-	-
After immersing into water									
9	(0.27/0.11)	0	180	- ^a^	-	-	-	-	-
10	(0.27/0.21)	0	180	- ^a^	-	-	-	-	-

^a^ No change.

**Table 5:** The atomic contents of carbon, oxygen, nitrogen, fluorine, and silicon of the modified PET fabric swatch before and after immersing into water.

	Atomic Contents (atm, %)				
	C	O	N	F	Si
Before immersing	39.9	33.1	20.1	6.3	0.6
After immersing	40.0	31.6	19.7	7.9	0.7

## Abbreviations

R_F_-(VM)*_n_*-R_F_	Fluoroalkyl end-capped vinyltrimethoxysilane oligomer
Glu-Si(OEt)_3_	*N*-(3-triethoxysilylpropyl)gluconamide
R_F_-(VM-SiO_3/2_)*_n_*-R_F_/Glu-SiO_3/2_	Fluoroalkyl end-capped vinyltrimethoxysilane oligomeric silica nanocomposites containing gluconamide units
PET	Polyethylene terephthalate
R_F_-(VM-SiO_3/2_)*_n_*-R_F_	Fluoroalkyl end-capped vinyltrimethoxysilane oligomeric silica nanoparticles
